# Prevalence and Molecular Characterization of Fluoroquinolone-Resistant *Escherichia coli* in Healthy Children

**DOI:** 10.3389/fcimb.2021.743390

**Published:** 2021-12-13

**Authors:** Qiang Zhao, Yueyun Shen, Gang Chen, Yanping Luo, Shenghui Cui, Yaping Tian

**Affiliations:** ^1^ Department of Laboratory Medicine, The First Medical Center, Chinese PLA General Hospital, Beijing, China; ^2^ Birth Defects Prevention and Control Technology Research Center, Chinese PLA General Hospital, Beijing, China; ^3^ Key Laboratory of Carcinogenesis and Translational Research (Ministry of Education/Beijing), Department of Clinical Laboratory, Peking University Cancer Hospital & Institute, Beijing, China; ^4^ Department of Food Science, National Institutes for Food and Drug Control, Beijing, China

**Keywords:** ESBL, *Escherichia coli*, fluoroquinolone, multidrug resistance, whole genome sequencing

## Abstract

Faecal *E. coli* can act as reservoirs for resistance genes. Here, we analyzed prevalence of drug resistance in faecal *E. coli* isolated from healthy children at a single kindergarten in Beijing, China, then used whole genome sequencing to characterize fluoroquinolone-non-susceptible strains. Our results revealed high resistance to ampicillin (54.0%), trimethoprim/sulphurmethoxazole (47.5%) and tetracycline (58.9%) among 576 faecal *E. coli* isolates, 49.2% of which exhibited multidrug resistance. A total of 113 *E. coli* isolates were not susceptible to ciprofloxacin, with four sequence types, namely ST1193 (25.7%), ST773 (13.3%), ST648 (8.8%) and ST131 (7.1%) found to be the most prevalent (54.9%). With regards to resistance to quinolones, we detected chromosomal mutations in *gyrA*, *parC*, and *parE* in 111 (98.2%), 105 (92.9%), and 67 (61.1%) isolates, respectively. *bla*_CTX-M_ (37.2%) was the major ESBL gene, whereas *bla*_CTX-M-14_ (12.4%) and *bla*_CTX-M-27_ (11.5%) were the most frequent subtypes. A total of 90 (79.6%) ExPEC and 65 (57.5%) UPEC isolates were classified. Overall, these findings revealed clonal spread of certain prevalent STs, namely ST1193, ST773, ST648 and ST131 *E. coli* isolates in healthy children within a single kindergarten in Beijing, China, affirming the seriousness of the multidrug resistance problem and potential pathogenicity of *E. coli* isolates in healthy children. Therefore, there is an urgent need for increased surveillance to enhance control of this problem.

## Introduction

*Escherichia coli* is an important foodborne opportunistic pathogen, that causes various extraintestinal infections, such as urinary tract infections and septicemia (Zhong et al., 2019; Bonten et al., 2021). Previous studies have reported a high prevalence of fluoroquinolone resistance and extended-spectrum β-lactamase (ESBL) production worldwide (Palma et al., 2017; Boll et al., 2020; Stapleton et al., 2020). In China, more than 50% of *E. coli* strains isolated from community-acquired infections are resistant to fluoroquinolones (Zhao et al., 2015), while 16% of these strains are reported to be ESBL-producing (Ling et al., 2006). Faecal *E. coli* can act as reservoirs for resistance genes, and are also considered a useful indicator for the spread of acquired antibiotic resistance genes in the community (Nys et al., 2004; Salyers et al., 2004). While geographical variation had been demonstrated in resistance rates of *E. coli* isolates in feces of healthy children (Sahoo et al., 2012), resistances to commonly used antibiotics like ampicillin, tetracycline and trimethoprim/sulfamethoxazole were frequently observed, especially in developing countries (Bartoloni et al., 2006; Dyar et al., 2012; Shakya et al., 2013; Ferjani et al., 2018; Mahmoodi et al., 2020). Over 90% of faecal samples from Chinese children tested positive for β-lactam, macrolide, tetracycline, and aminoglycoside resistance genes (Ravensdale et al., 2018), indicating the magnitude of the problem of antimicrobial drug resistance in China. To date, however, only a handful of reports have described antibiotic resistance in faecal *E. coli* among Chinese healthy children (Lester et al., 1990; Huang et al., 2018). In the present study, we focused on fluoroquinolone resistance in children, since it is restricted in the pediatric population due to concerns about significant adverse effects associated with its use (Jackson and Schutze, 2016). To this end, we determined prevalence of resistance genes in *E. coli* isolated from rectal swabs from healthy children in China, then applied whole genome sequencing to characterize fluoroquinolone-non-susceptible strains.

## Materials and Methods

### Bacterial Strains and Antimicrobial Susceptibility Testing

A total of 596 nonduplicate *E. coli* strains were isolated from rectal swab samples collected as part of routine physical examination from 736 children, aged between 3 and 6 years, at a kindergarten in Beijing, China, in October 2018. The strains were identified by matrix-assisted laser desorption/ionization time-of-flight mass spectrometry (MALDI-TOF MS) (Vitek MS; bioMérieux, France). The minimal inhibitory concentrations (MICs) of 10 antimicrobial agents, namely ampicillin, ampicillin/sulbactam, trimethoprim/sulfamethoxazole, ciprofloxacin, chloramphenicol, gentamicin, tetracycline, cefotaxime, ceftazidime, and imipenem, were assessed by the agar dilution method with *E. coli* strain ATCC 25922 as the control according to CLSI 2018. Multidrug resistance (MDR) was defined as resistance of an isolate to any antibiotic from at least three different antibiotic groups (Magiorakos et al., 2012).

### DNA Extraction and Whole Genome Sequencing

*E. coli* isolates resistant to ciprofloxacin were selected for whole genome sequencing. Briefly, genomic DNA was extracted from the isolates using a DNeasy Blood and Tissue Kit (Qiagen, Hilden, Germany), confirmed by agarose gel electrophoresis and quantified by a Qubit 4 Fluorometer (Thermo Fisher Scientific, Singapore, Singapore). Whole genome sequencing was performed on the Illumina HiSeq platform (Illumina, San Diego, CA, USA) to generate 2×150 bp pair-end reads. Raw reads were *de novo* assembled using SOAP (Li et al., 2008), and the genomes annotated using RAST v2.0 (Aziz et al., 2008) and prokka v1.12 (Seemann, 2014).

### Genome Analysis

Resistance genes, chromosomal mutations defining quinolone resistance, serotype, *fimH* subtype, multilocus sequence typing (MLST), and virulence genes of ciprofloxacin-resistant *E. coli* strains were analyzed using ResFinder 4.0, PointFinder, SerotypeFinder 2.0, FimTyper 1.0, MLST 2.0, and VirulenceFinder 2.0 tools, from the Center for Genomic Epidemiology (CGE) (https://cge.cbs.dtu.dk/services/). *E. coli* phylogenetic grouping was performed using the ClermonTyping tool (Beghain et al., 2018). Insertion sequence elements were identified using ISfinder (https://isfinder.biotoul.fr/). All drafts and ST-relevant genomes from Enterobase (Zhou et al., 2020) were submitted to kSNP3.0 (Gardner et al., 2015) for single nucleotide polymorphism (SNP) identification, then used to construct a phylogenetic tree with 100 bootstraps which was later visualized using the iTOL website (https://itol.embl.de/). Sequence comparison and map generation were performed using BLAST and Easyfig v2.1 (Sullivan et al., 2011), respectively. Strains were classified as extraintestinal pathogenic *E. coli* (ExPEC) if they exhibited positivity ≥2 for the following: *papAH* and/or *papC* (P fimbriae), *sfa-focDE* (S and F1C fimbriae), *afa-draBC* (Dr-binding adhesins), *iutA* (aerobactin siderophore system), and *kpsM II* (group 2 capsules). Strains were classified as uropathogenic *E. coli* (UPEC) if they showed a positivity ≥2 for *chuA* (heme uptake), *fyuA* (yersiniabactin siderophore system), *vat* (vacuolating toxin), and *yfcV* (adhesin) (Malberg et al., 2020).

### Statistical Analysis

Categorical variables were assessed using two-tailed chi-square or Fisher exact tests, where appropriate. Data followed by P ≤ 0.05 were considered statistically significant.

## Results

### Antimicrobial Susceptibility Testing

The results of resistant rates of individual antibiotics after antimicrobial susceptibility testing among the studied isolates are shown in **Table 1**. Summarily, a majority of the isolates were highly resistant to tetracycline (58.9%), followed by ampicillin (54.0%) and trimethoprim/sulphurmethoxazole (47.5%). On the other hand, low resistance was observed in the isolates to ceftazidime (0.3%), whereas no isolate was resistant to imipenem (0.0%). Almost half of the isolates (n= 293, 49.2%) were resistant to three or more different antibiotics, typical of multidrug resistance.

**Table 1:** MIC profiles of 10 antibiotics against 596 *Escherichia coli* strains isolated from rectal swab samples collected from 736 children.

Antimicrobial category	Antimicrobial agent	MIC (μg/ml)			% Resistant
		50%	90%	Range	
Aminoglycosides	Gentamicin	≤1	32	≤1-≥32	26.2
Penicillins	Ampicillin	64	≥64	≤2-≥64	54.0
Penicillins +β-lactamase inhibitors	Ampicillin/Sulbactam	8	32	≤2-≥64	12.6
Extended-spectrum cephalosporins	Ceftazidime	≤1	2	≤1-≥32	0.3
	Cefotaxime	≤0.25	≥8	≤0.25-≥8	16.3
Carbapenems	Imipenem	0.25	0.5	≤0.25-1	0.0
Fluoroquinolones	Ciprofloxacin	0.25	16	≤0.03-≥32	17.6
Tetracyclines	Tetracycline	≥32	≥32	≤1-≥32	58.9
Phenicols	Chloramphenicol	8	≥64	≤2-≥64	14.9
Folate pathway inhibitors	Trimethoprim/Sulfamethoxazole	0.5	≥8	≤0.25-≥8	47.5

### MLST and Phylogenetic Group

**Figure 1:**
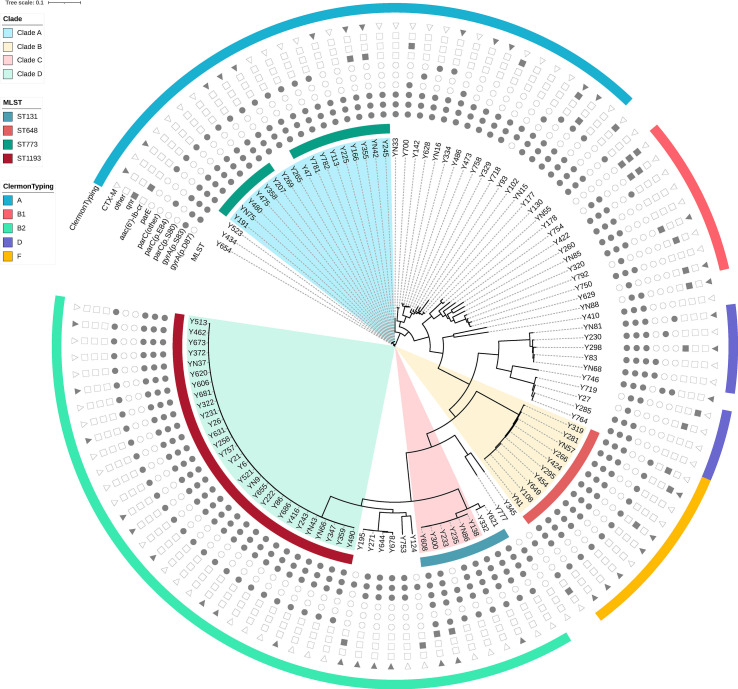
Phylogenetic tree and genomic characteristics of 113 *Escherichia coli* isolates. Solid and hollow signs indicate presence and absence of acquired resistance genes and chromosomal mutations in quinolone-resistance-determining-regions (QRDR), respectively.

A total of 113 ciprofloxacin-non-susceptible *E. coli* isolates were selected for whole genome sequencing. Phylogenetically, these strains were broadly distributed into the following groups: B2 (38.1%), A (31.0%), F (8.8%), B1 (8.0%), D (8.0%), G (2.7%), C (1.8%), and E (1.8%). The results from *in silico* MLST analysis revealed 35 different STs, including 3 novel ones (ST11494-11496) which have been submitted to Enterobase. ST1193 (25.7%), ST773 (13.3%), ST648 (8.8%) and ST131 (7.1%) were the most prevalent, accounting for 54.9% of the 113 *E. coli* isolates. Moreover, ST131 and ST1193 belonged to phylogenetic group B2, while ST648 and ST773 belonged to groups F and group A, respectively (**Figure 1**).

### Serogroup and *fimH* Type

Among 113 *E. coli* strains, 25 O serogroups and 20 *fimH* types were identified. The most prevalent O serogroup was O75 (24.8%), followed by O1 (13.3%), O25 (9.7%), and O21 (9.7%). In addition, *fimH* allele subtyping analysis showed that *fimH*64 was the most frequent subtype (25.7%), followed by *fimH*27 (9.7%), *fimH*54 (6.2%), and *fimH*30 (5.3%). Notably, almost all of the ST1193 isolates belonged to the O75-*fimH*64 type, while most of the ST648 and ST773 isolates belonged to O1 and O21 serogroups, respectively. For ST131 isolates, there were two major serogroup-*fimH* types, namely O25-*fimH*30 and O16-*fimH*41.

### Detection of Antimicrobial Resistance Genes

We adopted ResFinder and PointFinder tools for analysis of antimicrobial resistance genes, and detected resistance to eight groups, namely aminoglycosides, β-lactams, quinolones, macrolides, tetracyclines, phenicols, fosfomycin, and sulphonamides. Notably, all of the 113 ciprofloxacin-resistant *E. coli* strains carried one or more resistance genes, 103 (91.2%) out of which carried three or more resistances to different antibiotic groups. With regards to resistance to quinolones, we detected chromosomal mutations in *gyrA*, *parC*, and *parE* in 111 (98.2%), 105 (92.9%), and 67 (61.1%) isolates, respectively. The most prevalent chromosomal mutations included *gyrA* (p.S83L) (98.2%), *parC* (p.S80I) (90.3%), and *gyrA* (p.D87N) (81.4%). Moreover, the mutation rate of *parE* among the four most prevalent STs isolates (ST1193, ST773, ST648 and ST131) was significantly higher than that among other isolates (91.9% vs 23.5%, p=0.000). Notably, *parE* (p.L416F), *parE* (p.I529L) and *parE* (p.S458A) were the unique chromosomal mutation types of *parE* in ST1193, ST131 and ST648 isolates, respectively. Apart from chromosomal mutations, we also detected *aac (6’)-Ib-cr* (8.8%), *qnrS1* (7.1%), *oqxAB* (4.4%), *qnrS2* (2.7%) and *qnrB4* (1.8%). At least one candidate quinolone resistance gene or chromosomal mutation could be found in all of the 113 ciprofloxacin-resistant *E. coli* strains. With regards to *β*-lactam resistance, *bla*_CTX-M_ (37.2%) was the major ESBL gene, of which *bla*_CTX-M-14_ (12.4%) and *bla*_CTX-M-27_ (11.5%) were the most frequent subtypes. IS*Ecp1*/IS*Ecp1*Δ (100.0%) was found upstream of *bla*_CTX-M_ in both CTX-M-1 and CTX-M-9 groups. IS*903B*/IS*903B*Δ (100.0%) was always found downstream in the CTX-M-9 group, while *orf477*/*orf477*Δ (83.3%) was common in the CTX-M-1 group. Besides IS*Ecp1* and IS*903B*, IS*26* (57.1%) was also found to be adjacent to *bla*_CTX-M_ genes frequently (**Table 2**). For aminoglycoside resistance, the main genes carried by these isolates were *strA* (55.8%), *strB* (57.5%), *aadA5* (53.1%), and *aac(3)-IId* (40.7%). *sul1* (56.6%) and *sul2* (57.5%) were the most prevalent genes encoded sulphonamide resistance while *fosA3* (4.4%) and *fosA7* (1.8%) were the major genes for fosfomycin resistance. The main tetracycline resistance genes detected were *tet(A)* (61.9%) and *tet(B)* (17.7%). Neither *mcr* nor carbapenemase-encoding genes were detected.

**Table 2:** Genetic environments of *bla*_CTX-M_ in *Escherichia coli* isolates.

*bla*_CTX-M_		Genetic environment	No.
CTX-M-1 group	*bla*_CTX-M-3_	IS*26*-IS*Ecp1*Δ–*bla*_CTX-M-3_–IS*26*	2
		IS*26*-IS*Ecp1*Δ–*bla*_CTX-M-3_–*orf477*	2
		IS*Ecp1*–*bla*_CTX-M-3_–*orf477*Δ	1
	*bla*_CTX-M-15_	IS*26*-IS*Ecp1*Δ–*bla*_CTX-M-15_–*orf477*Δ-Tn*3*Δ-IS*26*	2
		IS*Ecp1*Δ–*bla*_CTX-M-15_–*orf477*Δ	1
	*bla*_CTX-M-55_	IS*26*-IS*Ecp1*Δ–*bla*_CTX-M-55_–*orf477*	1
		IS*Ecp1*Δ–*bla*_CTX-M-55_–*orf477*Δ	1
		IS*Ecp1*–*bla*_CTX-M-55_–*orf477*Δ	1
	*bla*_CTX-M-64_	IS*Ecp1*–*bla*_CTX-M-64_–*orf477*Δ	1
CTX-M-9 group	*bla*_CTX-M-14_	IS*Ecp1*–*bla*_CTX-M-14_–IS*903B*Δ	6
		IS*Ecp1*–*bla*_CTX-M-14_–IS*903B*	2
		IS*26*-IS*Ecp1*Δ–*bla*_CTX-M-14_–IS*903B*Δ	2
		IS*26*-IS*Ecp1*Δ–*bla*_CTX-M-14_–IS*903B*-IS*Ecp1*Δ	2
		IS*26*-IS*10*Δ-IS*Ecp1*Δ–*bla*_CTX-M-14_–IS*903B*	1
		IS*10*-IS*Ecp1*–*bla*_CTX-M-14_–IS*903B*Δ	1
	*bla*_CTX-M-27_	IS*26*-IS*Ecp1*Δ-*bla*_CTX-M-27_- IS*903B*	10
		IS*Ecp1*Δ-*bla*_CTX-M-27_- IS*903B*	2
		IS*26*-IS*Ecp1*Δ-*bla*_CTX-M-27_- IS*903B*Δ- IS*26*	1
	*bla*_CTX-M-65_	IS*Ecp1*Δ–*bla*_CTX-M-65_–IS*903B*	2
		IS*Ecp1*Δ–*bla*_CTX-M-65_–IS*903B*Δ-IS*26*	1

### Virulence Genes

The results from VirulenceFinder analysis revealed the presence of 90 (79.6%) ExPEC and 65 (57.5%) UPEC isolates among the 113 ciprofloxacin-resistant *E. coli* strains. All of the ST131 and ST1193 isolates, which belonged to phylogenetic group B2, could be classified as both ExPEC and UPEC owing to the fact that they harbored *iutA, kpsMII/kpsMII_K1/kpsMII_K5, papA, chuA*, and *fyuA* virulence genes. Despite belonging to phylogenetic group A, and different from ST131 and ST1193, all 15 ST773 isolates were identified as ExPEC, and harbored *kpsMII_K1, papA*, and *papC* virulence genes (**Table 3**).

**Table 3:** Distribution of virulence genes associated with ExPEC/UPEC identification in 113 ciprofloxacin-non-susceptible *Escherichia coli* isolates (n, %).

Virulence factor	non-prevalent STs		ST1193B2 n = 29	ST131B2 n = 8	ST648F n = 10	ST773A n = 15	Total n = 113
	B2/D n = 15	Other n = 36					
ExPEC	14 (93.3)	17 (47.2)	29 (100.0)	8 (100.0)	7 (70.0)	15 (100.0)	90 (79.6)
*iutA*	13 (86.7)	23 (63.9)	29 (100.0)	8 (100.0)	7 (70.0)	7 (46.7)	87 (77.0)
*afaC*	3 (20.0)	0 (0.0)	0 (0.0)	0 (0.0)	2 (20.0)	0 (0.0)	5 (4.4)
*nfaE*	3 (20.0)	0 (0.0)	0 (0.0)	0 (0.0)	2 (20.0)	0 (0.0)	5 (4.4)
*kpsMII*	6 (40.0)	4 (11.1)	0 (0.0)	2 (25.0)	1 (10.0)	0 (0.0)	13 (11.5)
*kpsMII_K1*	5 (33.3)	2 (5.6)	29 (100.0)	0 (0.0)	0 (0.0)	15 (100.0)	51 (45.1)
*kpsMII_K5*	4 (26.7)	3 (8.3)	0 (0.0)	6 (75.0)	8 (80.0)	0 (0.0)	21 (18.6)
*kpsMII_K52*	0 (0.0)	1 (2.8)	0 (0.0)	0 (0.0)	0 (0.0)	0 (0.0)	1 (0.9)
*papA*	12 (80.0)	15 (41.7)	29 (100.0)	8 (100.0)	0 (0.0)	15 (100.0)	79 (69.9)
*papC*	6 (40.0)	12 (33.3)	0 (0.0)	2 (25.0)	0 (0.0)	15 (100.0)	35 (31.0)
*focC/sfaE*	0 (0.0)	1 (2.8)	0 (0.0)	0 (0.0)	0 (0.0)	0 (0.0)	1 (0.9)
*sfaD*	0 (0.0)	1 (2.8)	0 (0.0)	0 (0.0)	0 (0.0)	0 (0.0)	1 (0.9)
UPEC	14 (93.3)	4 (11.1)	29 (100.0)	8 (100.0)	10 (100.0)	0 (0.0)	65 (57.5)
*chuA*	15 (100.0)	5 (13.9)	29 (100.0)	8 (100.0)	10 (100.0)	0 (0.0)	67 (59.3)
*fyuA*	13 (86.7)	19 (52.8)	29 (100.0)	8 (100.0)	10 (100.0)	14 (93.3)	93 (82.3)
*vat*	6 (40.0)	1 (2.8)	28 (96.6)	0 (0.0)	0 (0.0)	0 (0.0)	35 (31.0)
*yfcV*	6 (40.0)	0 (0.0)	29 (100.0)	7 (87.5)	10 (100.0)	0 (0.0)	52 (46.0)

### Prevalence of *E. coli* ST773 Isolates

**Figure 2:**
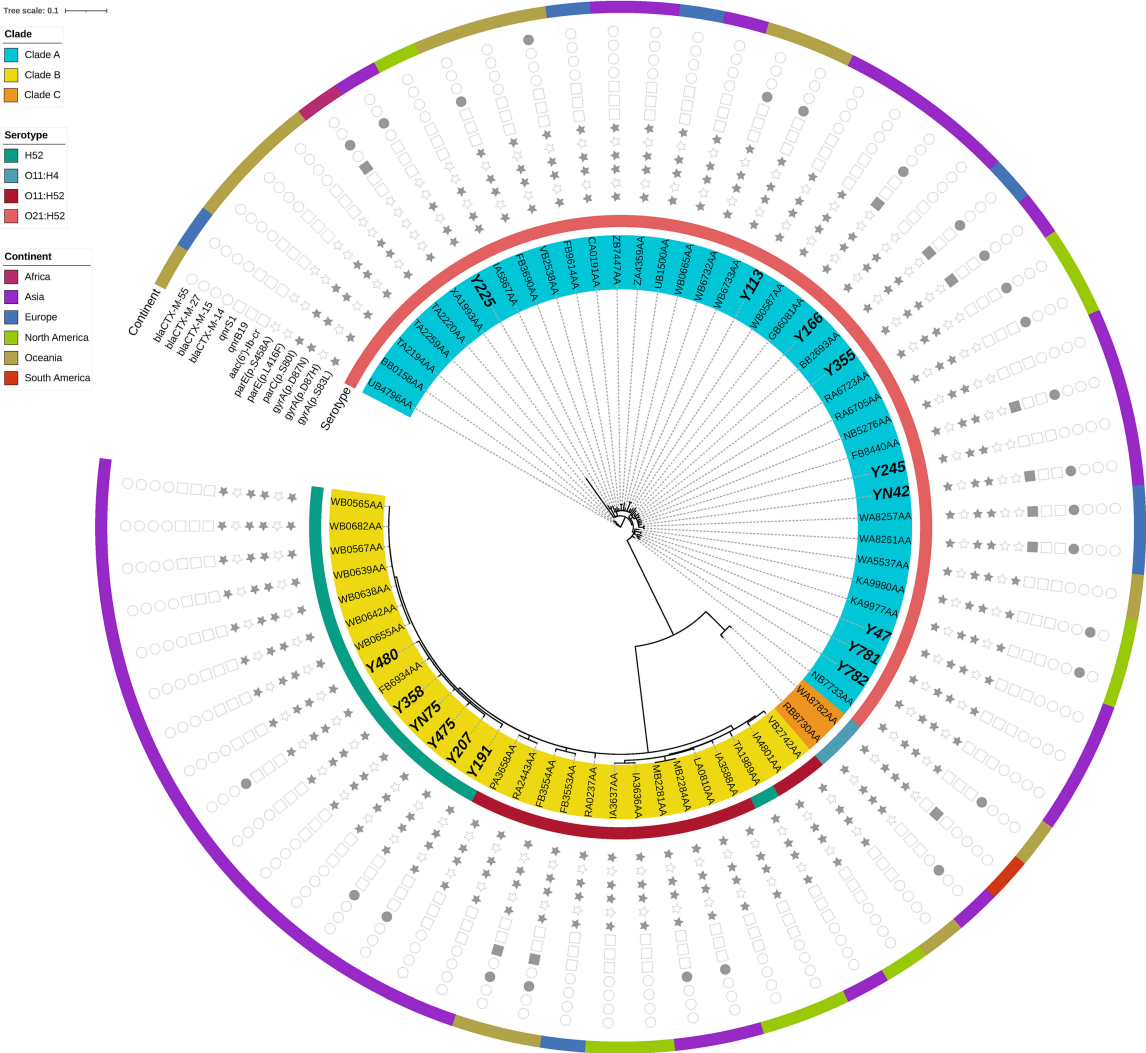
Phylogenetic tree and genomic characteristics of 51 *Escherichia coli* ST773 isolates. Solid and hollow signs indicate presence and absence of acquired resistance genes and chromosomal mutations in QRDR, respectively.

The phylogenetic tree obtained by core-genome SNP analysis corroborated the findings from MLST analysis and ClermonTyping phylogenetic group segregation (A, B1, B2, C, D, E, and F). Notably, the four most prevalent STs, ST1193, ST773, ST648 and ST131, were clustered within a monophyletic clade (**Figure 1**). We also downloaded sequences of draft genomes of 54 ST773 strains isolated across 6 continents (Africa, Asia, Europe, North America, Oceania, and South America) from Enterobase, and incorporated them in our genome analysis to construct a phylogenetic tree comprising 69 ST773 isolates. The results showed that all ST773 isolates belonged to the phylogenetic group A, and revealed 4 serogroups. The most prevalent serogroup was O21:H52 (56.5%), followed by H52 (21.7%), O11:H52 (18.8%), and O11:H4 (2.9%). The phylogenetic tree further clustered the four serogroup isolates into three clades, designated A-C: O11:H52 and H52 serogroups belonged to clade B while O21:H52 serogroup belonged to clade A (**Figure 2**). Notably, we detected the same chromosomal mutations in all but three of the ST773 isolates, namely *gyrA* (p.S83L)-*gyrA* (p.D87N)-*parC* (p.S80I). In addition, the chromosomal mutation rate of *parE* (p.S458A) in O11:H52 and H52 isolates (clade B) was significantly higher than that of O21:H52 isolates (clade A) (100.0% vs 38.7%, p=0.000). With regards to resistance to β-lactams, *bla*_CTX-M_ (37.7%) was the major ESBL gene, of which *bla*_CTX-M-14_ (26.1%) and *bla*_CTX-M-15_ (7.2%) were the most frequent types.

## Discussion

Antibiotic resistance is a major global public health concern. Faecal *E. coli* is considered a key indicator for the transmission of acquired antibiotic resistance genes in the community. For instance, a previous systematic review reported that resistance to many primary care prescribed antibiotics was common among *E. coli* from faecal matter, and this was carried by asymptomatic children, especially in non-Organization for Economic Co-operation and Development countries (Bryce et al., 2016). In addition, multidrug resistance and ESBL production of commensal *E. coli* isolates were observed in more than 36.2 and 11.7%, respectively, among healthy children under 3 years old in Iran (Mahmoodi et al., 2020). The results from the present study revealed that a majority of the *E. coli* strains isolated from faecal matter of Chinese healthy children were highly resistant to ampicillin, trimethoprim/sulphurmethoxazole and tetracycline, while almost half of the isolates were multidrug resistant, which was consistent with previous studies (Bartoloni et al., 2006; Shakya et al., 2013; Mahmoodi et al., 2020). Notably, although the use of fluoroquinolones is restricted in the pediatric population because of the associated musculoskeletal adverse effects (Jackson and Schutze, 2016), 17.6% of the *E. coli* strains exhibited resistance to ciprofloxacin in this study.

Next, we employed whole genome sequencing to determine genetic characteristics of the 113 ciprofloxacin-resistant *E. coli* strains. Overall, the results indicated that resistance to fluoroquinolones was associated with amino acid substitutions in quinolone-resistance-determining-regions (QRDR), including *gyrA*, *parC*, and *parE*. *bla*_CTX-M_, especially *bla*_CTX-M-14_ and *bla*_CTX-M-27_, contributed to third-generation cephalosporin resistance. In addition, more than 90% of those strains harbored resistance genes for three or more antibiotic groups, while 80% and 60% of them were classified as ExPEC and UPEC strains, respectively. These findings affirmed the seriousness of the problem of multidrug resistance and potential pathogenicity of *E. coli* isolates in healthy children. Therefore, there is an urgent need for increased surveillance and control of this phenomenon.

The results from our phylogenetic and MLST analyses revealed existence of clonal spread of *E. coli* isolates, especially for the four most prevalent STs, namely ST1193, ST773, ST648 and ST131, consistent with previous studies that have demonstrated that *E. coli* strains can be shared within households (Madigan et al., 2015; Johnson et al., 2016). In fact, *E. coli* ST131 has emerged as a major pathogen of blood-stream and urinary tract infections worldwide (Chen et al., 2019; Birgy et al., 2020; Findlay et al., 2020; Holland et al., 2020). To date, several factors, including resistance to fluoroquinolones, affiliation to phylogroup B2, and high virulence gene contents, have been associated with its successful spread (Valenza et al., 2019). Petty et al. reported that most fluoroquinolone-resistant ST131 strains belonged to a single subclone, designated clade C. Surprisingly, most CTX-M-15-producing ST131 isolates were also derived from a single clade within clade C, named clade C2 (Petty et al., 2014). In the present study, we identified 8 ST131 strains among the 113 ciprofloxacin-resistant *E. coli* isolates, of which 2 were CTX-M-15 positive. Notably, 6 of the 8 ST131 isolates clustered in clade C (**Figure S1**), while the 2 CTX-M-15-producing ST131 belonged to clade C2 (**Figure S2**), which was consistent with the findings of Petty et al. (2014). Since 2012, a new fluoroquinolone-resistant clone namely ST1193, and belonging to phylogenetic group B2, has been reported worldwide (Zhao et al., 2015; Johnson et al., 2019; Tchesnokova et al., 2019). Results of the present study showed that ST1193 was one of the most prevalent STs, accounting for a quarter of all ciprofloxacin-resistant strains. Almost all of the ST1193 isolates belonged to the O75-*fimH*64 type, and exhibited a set of four conserved mutations in QRDR (*gyrA* S83L, *gyrA* D87N, *parC* S80I and *parE* L416F), which was consistent with the findings of Valenza et al. (2019). ST648 strains have been reported globally in human patients and more incidentally from animals, because of ESBL phenotype and ExPEC-association (Ewers et al., 2014; Fernandes et al., 2018). Apart from the well-known prevalence of ST131, ST1193 and ST648, ST773 has been considered another key ST, despite having been rarely reported in the past. Despite its affiliation with phylogenetic group A, all 15 ST773 isolates were identified as ExPEC. In fact, almost all of these isolates harbored three conserved mutations that were associated with fluoroquinolone resistance (*gyrA* D87N, *gyrA* S83L, *parC* S80I), while nearly one-third of the isolates carried the *bla*_CTX-M_ gene. Our phylogenetic tree revealed that 36 ST773 strains isolated across 6 continents exhibited a high degree of homology with our isolates, suggesting the potential of the widespread dissemination of this clone.

In conclusion, we identified four prevalent STs, namely ST1193, ST773, ST648 and ST131, in *E. coli* isolates. These exhibited clonal transmission across healthy children within a single kindergarten in Beijing, China. Since multidrug resistance and potential pathogenicity of *E. coli* is a serious problem among healthy children, we envisage that our findings will stimulate relevant discussions to guide urgent development of surveillance approaches to help control this phenomenon.

## Data Availability Statement

The datasets generated for this study can be found in GenBank under BioProject no. PRJNA679380.

## Ethics Statement

Neither ethics committee approval, nor informed consent were required as all collected data was fully anonymized, there was no contact with patients and/or their families and no interventions to treatment were made, in accordance with local guidelines.

## Author Contributions

QZ, YL, SC, and YT conceived and designed the study. YS, GC, and YL acquired the data. QZ drafted the manuscript. SC and YT critically revised the manuscript. All authors contributed to manuscript revision, read, and approved the submitted version.

## Funding

This work was supported by funds from the National Key Research and Development Program of China (Grant number: 2017YFC1601400).

## Conflict of Interest

The authors declare that the research was conducted in the absence of any commercial or financial relationships that could be construed as a potential conflict of interest.

## Publisher’s Note

All claims expressed in this article are solely those of the authors and do not necessarily represent those of their affiliated organizations, or those of the publisher, the editors and the reviewers. Any product that may be evaluated in this article, or claim that may be made by its manufacturer, is not guaranteed or endorsed by the publisher.

## Supplementary Material

Supplementary Figure 1 Phylogenetic tree and genomic characteristics of 103 *Escherichia coli* ST131 isolates, including 8 isolates (in bold italics) reported in this study alongside 95 isolates reported by Petty et al. (https://github.com/BeatsonLab-MicrobialGenomics/ST131_99). Solid and hollow signs indicate presence and absence of the acquired resistance genes and chromosomal mutations in QRDR, respectively.

Supplementary Figure 2 Phylogenetic tree and genomic characteristics of 71 *Escherichia coli* ST131 isolates that belonged to C clade, including 6 isolates (highlighted by bold italic title) reported in this study alongside 65 isolates reported by Petty et al. (https://github.com/BeatsonLab-MicrobialGenomics/ST131_99).

Supplementary Table 1 MIC of 10 antibiotics against 596 *Escherichia coli* strains isolated from rectal swab samples collected from 736 children.

Supplementary Table 2 Distribution of antimicrobial resistance genes and virulence genes in 113 ciprofloxacin-non-susceptible *Escherichia coli* isolates.

Supplementary Table 3 Distribution of antimicrobial resistance genes and virulence genes in 69 *Escherichia coli* ST773 isolates.
