# Supplementary figures and images for: Prevalence and Molecular Characterization of Fluoroquinolone-Resistant Escherichia coli in Healthy Children
Source: Front Cell Infect Microbiol. 2021 Dec 13;11:743390. doi: 10.3389/fcimb.2021.743390 (PMC8710580; doi:10.3389/fcimb.2021.743390)

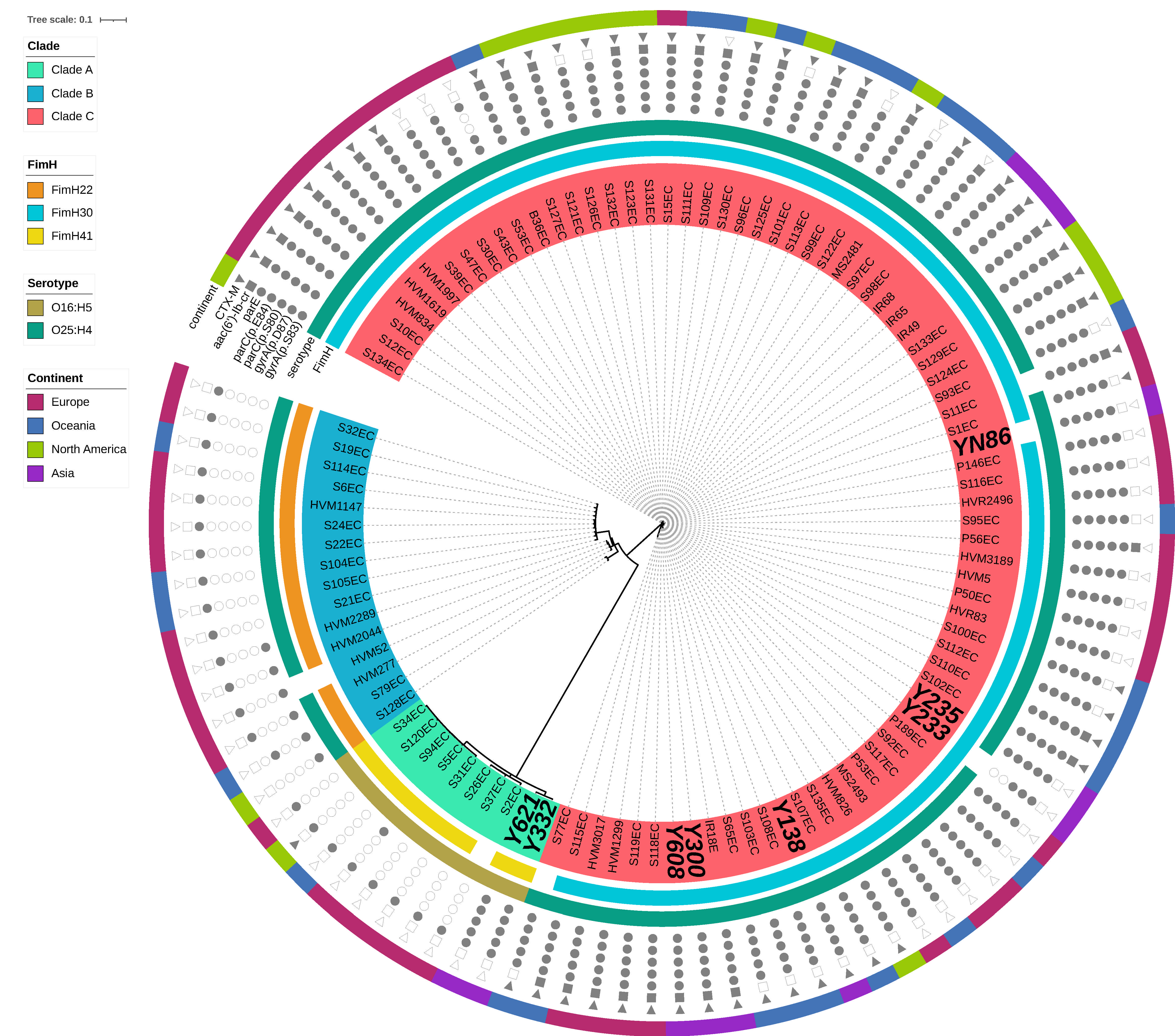

Supplement: Supplementary Figure 1 — Phylogenetic tree and genomic characteristics of 103 Escherichia coli ST131 isolates, including 8 isolates (in bold italics) reported in this study alongside 95 isolates reported by Petty et al. (https://github.com/BeatsonLab-MicrobialGenomics/ST131_99). Solid and hollow signs indicate presence and absence of the acquired resistance genes and chromosomal mutations in QRDR, respectively. [file Image_1.tiff]

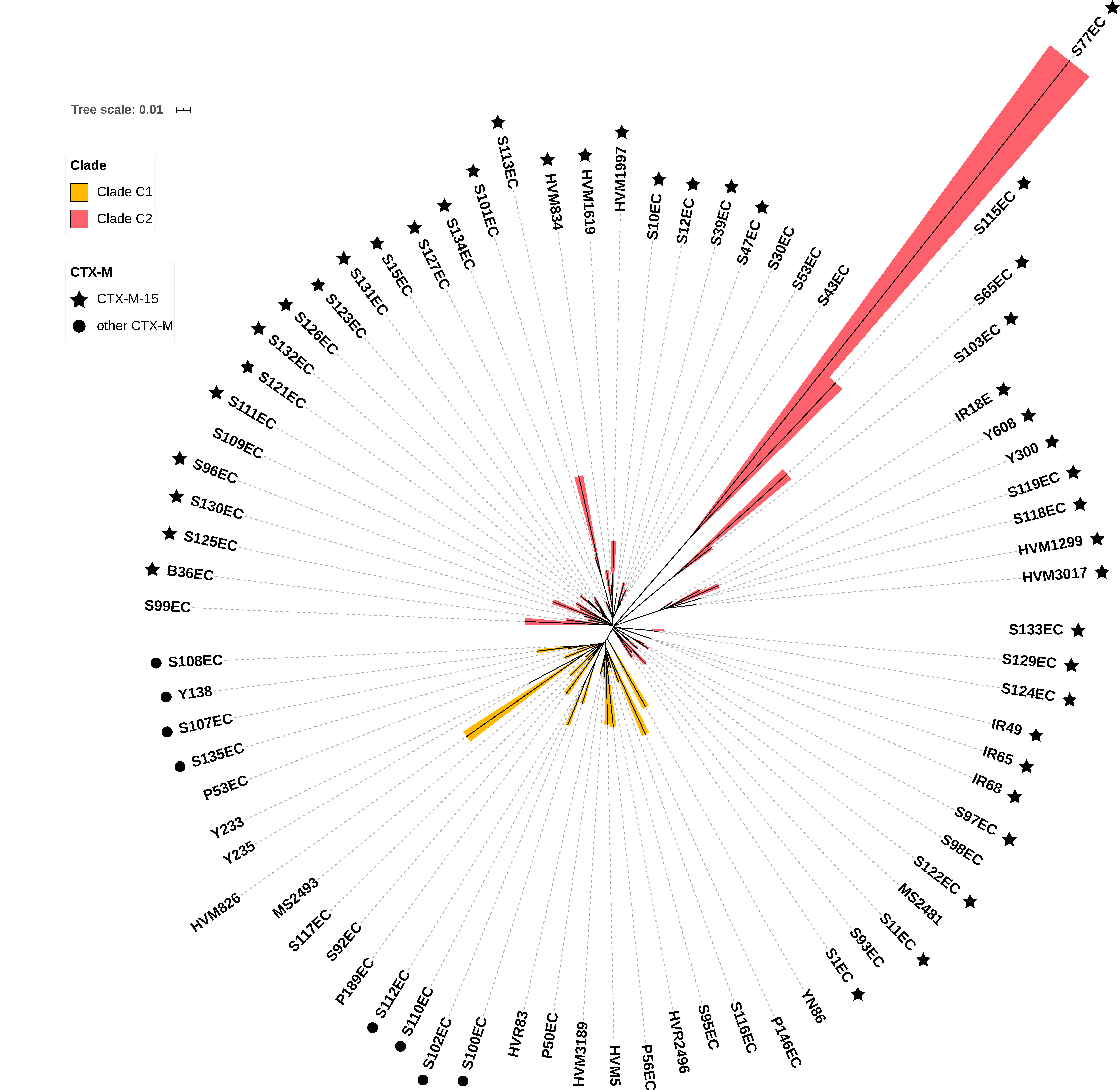

Supplement: Supplementary Figure 2 — Phylogenetic tree and genomic characteristics of 71 Escherichia coli ST131 isolates that belonged to C clade, including 6 isolates (highlighted by bold italic title) reported in this study alongside 65 isolates reported by Petty et al. (https://github.com/BeatsonLab-MicrobialGenomics/ST131_99). [file Image_2.tiff]
